# Synthesis of Nano-Scale Biopolymer Particles from Legume Protein Isolates and Carrageenan

**DOI:** 10.17113/ftb.58.02.20.6279

**Published:** 2020-06

**Authors:** Indika Dilrukshi Koralegedara, Charith Aravinda Hettiarachchi, Batugahage Don Rohitha Prasantha, Kuruppu Mudiyanselage Swarna Wimalasiri

**Affiliations:** Department of Food Science and Technology, Faculty of Agriculture, University of Peradeniya, 20400 Peradeniya, Sri Lanka

**Keywords:** biopolymer particles, protein-polysaccharide interactions, electrostatic complexes, legume proteins, carrageenan

## Abstract

**Research background:**

Food proteins and polysaccharides can be used for the synthesis of nano-scale biopolymer particles with potential applications in the fields of food and pharmaceuticals. This study focuses on utilizing legume proteins for the production of biopolymer particles *via* regulation of their electrostatic interactions with carrageenan.

**Experimental approach:**

Protein isolates were obtained from mung bean (*Vigna radiata*), cowpea (*Vigna unguiculata*) and black gram (*Vigna mungo*) and their protein profiles were determined. Next, these isolates were allowed to interact with carrageenan at pH=5.0-7.0 to determine optimum conditions for obtaining nano-scale biopolymer particles. Selected biopolymer mixtures were then subjected to a heat treatment (85 °C for 20 min) to enhance the interactions among biopolymers.

**Results and conclusion:**

Nano-scale biopolymer complexes were obtained at pH=6.5. They were roughly spherical in shape with a majority having a diameter in the range of approx. 100-150 nm. Heating of the biopolymer mixtures increased the diameter of the biopolymer particles by approx. 2.5-fold. In addition, their negative surface charge was increased, stabilizing them against aggregation over a broader pH range (4.0-7.0), enhancing their potential to be utilized in food matrices.

**Novelty and scientific contribution:**

This study reports the applicability of mung bean, cowpea and black gram proteins for the synthesis of stable biopolymer particles. These biopolymer particles can be potentially used for the encapsulation and delivery of bioactive components.

## INTRODUCTION

Formation of nano-scale particles from edible biopolymers has gained considerable interest due to their applicability in pharmaceuticals and processed foods as delivery agents of bioactive compounds *via* adsorption or encapsulation (*1*-*5*). Moreover, certain studies have shown that such particles can mimic rheological, optical and sensory properties of fat, enabling them to be used as fat replacers (*6*, *7*).

Food proteins and anionic polysaccharides have become common candidates for the fabrication of these biopolymer particles, because they carry opposite charges at food related pH values, enabling them to form electrostatic complexes. The extent of electrostatic interactions can be regulated relatively easily by adjusting the pH and/or ionic strength of the interaction medium, enabling a higher degree of control (*8*, *9*). At pH values below the isoelectric point (pI) of the proteins, where the proteins carry a net positive charge, extensive complex formation takes place between proteins and anionic polysaccharides resulting in a liquid coacervate phase or macroscopic aggregates, which usually result in a precipitate in the mixture (*8*). However, at pH values slightly above the pI of the proteins, where the proteins carry a net negative surface charge, they form soluble complexes with anionic polysaccharides due to the limited electrostatic interactions with positively charged sites still available on the protein surface (*9*, *10*). Stability of these biopolymer particles against aggregation can be enhanced by increasing the ionic strength of the medium or their surface charge (*11*).

There are reports on the formation of biopolymer particles by electrostatic complexation between β-lactoglobulin and pectin (*12*-*14*), β-lactoglobulin and sodium alginate (*15*), lactoferrin and carrageenan (*16*), whey protein isolate and sodium alginate (*17*), sodium caseinate and gum Arabic (*18*), and gelatin and gum Arabic, cress seed gum and zedo gum (*19*, *20*). However, only a few studies have been conducted to form biopolymer particles using plant proteins (*21*-*23*).

Due to the higher protein content in seeds (cereals, legumes and oil seeds; approx. 20-45% *m*/*m*) (*24*-*26*), they have become the popular choice among the sources of plant proteins. Seed proteins, based on their solubility, can be categorized into three major types, namely, albumins, globulins and prolamins (*27*). Globulins are the predominant type of protein found in a vast variety of legume seeds, and based on their sedimentation coefficients, they are further classified into 7S vicilins and 11S legumins (*28*, *29*). In general, vicilins are trimeric proteins with a molecular mass of approx. 150 kDa made with subunits of approx. 50 kDa. Legumins are made of six subunit pairs and each pair is made of approx. 20 kDa and approx. 40 kDa subunits, which are linked *via* a disulfide bond (*27*). Thus, legumins have a higher molecular mass than vicilins.

Due to the vast array of potential applications of biopolymer particles, as stated above, it would be worthwhile to investigate different sources of legume proteins that can be used for this purpose. During this study, three legume varieties rich in proteins, namely, mung bean, cowpea and black gram were selected as the protein source for the formation of biopolymer particles. The main objectives of this study are to form biopolymer particles by manipulating electrostatic interactions between the selected legume proteins and carrageenan, and to study the effect of thermal treatment on the pH stability and morphology of fabricated biopolymer particles.

## MATERIALS AND METHODS

### Materials

Three types of legumes, namely, mung bean (*Vigna radiata*), cowpea (*Vigna unguiculata*) and black gram (*Vigna mungo*) were selected for this study. Mung bean variety MI6, cowpea variety MI35 and black gram variety MI1 were purchased from the seed farm of the Department of Agriculture, Pelwehera, Sri Lanka. All these seeds had been pre-cleaned and they were free of debris and foreign materials. A sample of carrageenan, predominantly of the iota class was obtained from Motha Confectionery Works (Pvt) Ltd., Colombo, Sri Lanka. Chemicals used for the extraction of proteins and determination of protein content were obtained from VWR International (Darmstadt, Germany) and Sigma-Aldrich, Merck (St. Louis, MO, USA). Chemicals used for gel electrophoresis were purchased from Promega Corporation (Madison, WI, USA) and MP Biomedicals (Irvine, CA, USA).

### Preparation of legume seed flour samples

For the preparation of legume seed flour samples, approx. 200 g of respective seed varieties were ground using a high-speed mill with a removable metal sample cup (Stein M2; Steinlite Corporation, Atchison, KS, USA). Before milling, the removable metal cup (with the sample) was cooled by immersing in ice, and milling was performed in short intervals (approx. 5 s, 3-4 times) to prevent heat accumulation that may cause denaturation of proteins. The ground seed samples were sieved using a 200-μm mesh (Haver Analysensiebe, Oelde, Germany). Crude protein content of all the legume seed flour samples was determined by Kjeldahl method in triplicate (*30*). Nitrogen conversion factor used for the calculations was 5.6 (*31*). The protein mass fraction of the selected varieties of mung bean, cowpea and black gram was (26.6±0.9), (25.4±0.1) and (26.9±0.9) %, respectively.

Legume seed flour samples were then defatted by cold extraction to increase the efficiency of protein extraction. For the cold extraction, legume seed flour was mixed with hexane at a ratio of 50% (*m*/*V*) and stirred continuously at 150 rpm using a magnetic stirrer (SB 162; Stuart, Stone, UK) for 1.5 h. Next, the hexane was carefully decanted, residue flour sample was spread on a muslin cloth and allowed to dry for 24 h in an air circulating fume hood to remove the remaining hexane. The obtained defatted legume seed flour (further in text: defatted flour) samples were stored at 4 °C in sealed containers until they were further used.

### Protein extraction

Protein extraction was carried out according to the method described by Boye *et al*. (*32*). A dispersion (10%, *m*/*V*) was prepared from each defatted flour and its pH was adjusted to 8.5 with 0.1 M NaOH using a pH meter (Ecosan pH5; Eutech, Selangor, Malaysia), which is calibrated daily before usage. The pH adjusted dispersion was stirred for 1 h at ambient temperature ((28±2) °C) using the magnetic stirrer, and then it was centrifuged at 2200×*g* for 15 min (CT-4D; Hitachi, Tokyo, Japan). The supernatant was obtained and its pH was adjusted to 4.6 using 0.1 M HCl. Next, it was stirred for 1 h at ambient temperature and then it was centrifuged as mentioned earlier. The supernatant was discarded and the retentate was suspended in water. Next, the pH of the suspension was adjusted to 7.0 using 0.1 M NaOH, and it was dialyzed against water for 48 h at 4 °C using a 3.5 kDa molecular mass cut-off membrane. The dialyzed solution was then lyophilized (Lyotrap-Plus; LTE Scientific Ltd., Oldham, UK), and the obtained legume protein isolate (LPI) samples were stored at 4 °C in sealed containers until they were used.

### Estimation of protein yield

Crude protein content of the defatted flour and LPI samples was measured using the Kjeldahl method (*30*) in triplicate. The percentage of protein yield from defatted legume seed flour (DLSF) was calculated using the following equation (*33*):


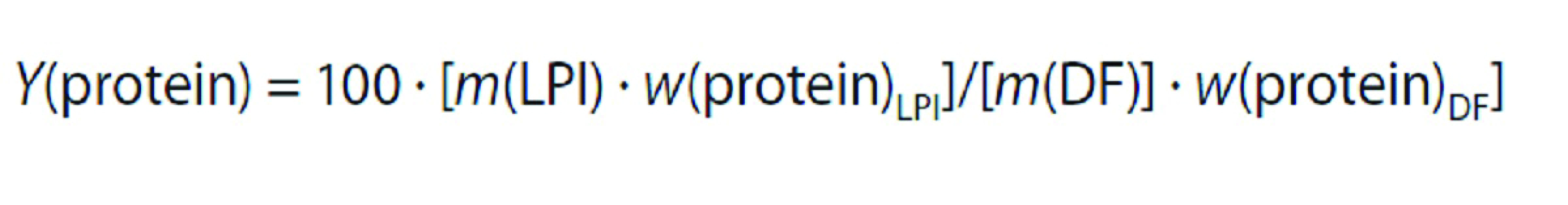


where *Y*(protein) is protein yield, *m*(LPI) is mass of the obtained LPI, *w*(protein)_LPI_ is protein mass fraction in the LPI, *m*(DF) is mass of defatted flour and *w*(protein)_DF_ is the mass fraction of proteins in the defatted flour. Mass of the flour sample taken for the determination of protein yield was 45.0 g.

### Protein profiling of LPI samples

Sodium dodecyl sulfate polyacrylamide gel electrophoresis (SDS-PAGE) was conducted to determine the molecular mass of the proteins and peptides present in the LPI samples (*34*). Electrophoresis was conducted using a non-gradient polyacrylamide gel casted in the laboratory, and it consisted of a 4% stacking gel and a 12% resolving gel. Lyophilized LPI samples were dissolved in water to obtain 3% (*m*/*V*) solution, and then further diluted to 1:2 (*V*/*V*) with Tris-HCl (pH=6.8) sample buffer, which contained 5% (*V*/*V*) β-mercaptoethanol. The samples were heated at approx. 95 °C for 4 min in a water bath and an aliquot of 12 μL from each sample was loaded into the wells of the polyacrylamide gel. A broad range molecular mass marker (10-225 kDa) was also loaded for the identification of molecular mass of the resulting sample bands. The gel was electrophoresed at 150 V, and then it was stained with a solution of 0.2% (*m*/*V*) Coomassie brilliant blue dissolved in 10% (*V*/*V*) acetic acid and 40% (*V*/*V*) methanol for about an hour. Finally, the gel was destained using a solution of 5% (*V*/*V*) acetic acid and 20% (*V*/*V*) methanol for 24 h.

### Preparation of LPI and carrageenan solutions

LPI solutions (0.1 and 0.2%, *m*/*V*) were prepared by dissolving lyophilized LPI powders in water. The solutions were stirred for 30 min at ambient temperature for the complete hydration of the powders. A carrageenan solution 0.1% (*m*/*V*) was prepared by dissolving carrageenan in hot water. The solution was stirred for 30 min at 80 °C to ensure complete hydration. Next, LPI and carrageenan solutions were centrifuged at 1100×*g* (CT-4D; Hitachi) for 15 min to remove any remaining insoluble materials and their pH was adjusted to 7.0 using 0.1 M NaOH. The contents of LPI and carrageenan given in the text are not corrected for minor changes that can occur due to centrifugation and pH adjustment.

### Formation of biopolymer particles

LPI and carrageenan solutions, 0.2 and 0.1% (*m*/*V*), respectively, at pH=7, were mixed in equal volumes to allow interactions between legume proteins and carrageenan molecules. These mixtures were kept for 1 h at ambient temperature before they were subjected to further analysis. The pH of these mixtures was verified to be 7.0±0.2. In order to manipulate the electrostatic interactions between the two biopolymers, the pH of the mixtures was gradually adjusted to values below 7.0 (up to pH=4.0) using 0.1 M HCl. These mixtures were also kept for 1 h at ambient temperature before they were subjected to further analysis.

### ζ-Potential measurements

For *ζ*-potential measurements, aliquots (10 mL) obtained from 0.1% (*m*/*V*) LPI and carrageenan solutions at pH=7.0, were adjusted to required pH values using 0.1 M HCl, and they were kept for 1 h at ambient temperature. A dynamic light scattering device (Nano-ZS; Malvern Instruments, Malvern, UK) was used for the measurements. This device determines the surface charge by tracing the electrophoretic mobility of the constituents present in the solution under the applied potential difference, and then converts it to the *ζ*-potential using the following equation (*35*):
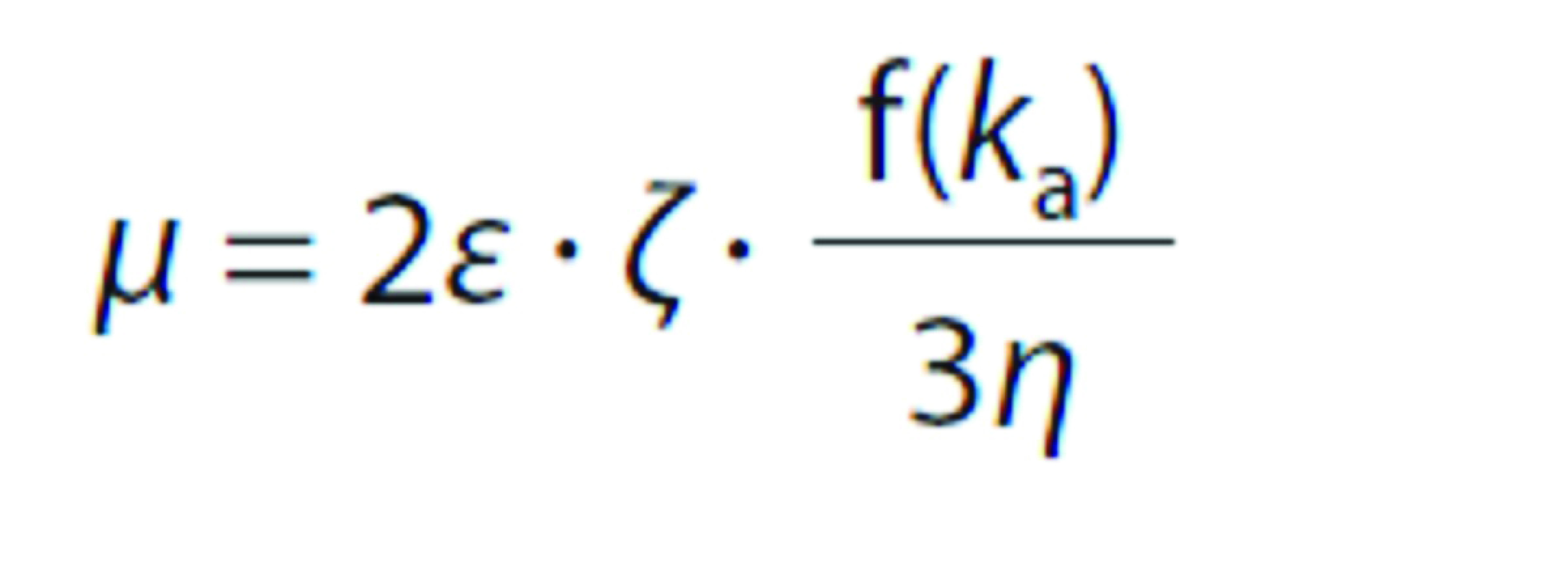
where *µ* is electrophoretic mobility, *ζ* is zeta potential, f(*k*_a_) is Henry’s function (taken as 1.5 according to Smoluchowski approximation), *ε* and *η* are dielectric constant and viscosity of the medium, respectively, which were assumed to be the same as those of water due to the very diluted solutions used. The cell used for the measurements was filled with the sample with a syringe without trapping any air bubbles. In addition to individual biopolymer solutions, *ζ*-potential of the mixtures made at pH=6.5 was also measured before and after the heat treatment.

### Transmittance measurements

Formation of biopolymer particles (large enough to scatter light) in the mixtures and their stability against precipitation at different pH values were monitored by measuring transmittance at 600 nm using a spectrophotometer (UV-1650; Shimadzu, Tokyo, Japan). No absorption occurs from the constituents present in the mixtures at the selected wavelength. A quartz cuvette with a path length of 1 cm was used for the measurements. Transmittance of individual biopolymer solutions at different pH values was also measured as control. The magnitude of reduction in transmitted intensity is considered to be inversely proportional to the level of interactions among biopolymers.

### Heat treatment of biopolymer particles

A volume of 20 mL obtained from each mixture at pH=6.5 was placed inside a screw-capped glass vial and heated in a water bath set at 85 °C for 20 min. The heat-treated mixtures were allowed to reach ambient temperature and they were diluted fivefold with water to ensure adequate transmittance of light. Aliquots (10 mL) obtained from these diluted mixtures were adjusted to different pH values between 4.0-7.0 with 0.1 M HCl or 0.1 M NaOH and kept for 1 h at ambient temperature. Transmittance of these heat-treated mixtures was measured as described above.

### Scanning electron microscopy

Morphology of the biopolymer particles formed in the mixtures at pH=6.5 before and after the heat treatment was observed using a scanning electron microscope (SEM; EVO; Carl-Zeiss, Cambridge, UK). An aliquot (10 μL) pipetted out from each mixture was deposited onto a carbon tape that had been fixed to a SEM stub, and then it was dried using a stream of air. The dried samples were gold coated using a sputter coater (SE7620; Quorum, Lewes, UK) and observed using the microscope at 10 kV. Individual biopolymer solutions were also subjected to SEM as controls. Diameter of the biopolymer particles observed by SEM was measured using imageJ software, v. 1.52a (*36*), from which the pixels were converted to nm using the scale bar of each image.

### Statistical analyses

Data acquisition was done on experiments carried out in triplicate. Significant differences between samples were assessed at a p=0.05 level by either one-way analysis of variance (ANOVA) or paired T-test. When ANOVA was performed, mean comparison was done using Tukey’s test.

## RESULTS AND DISCUSSION

### Protein yield of LPI samples

Table 1 presents the crude protein content of defatted flour and LPI samples, and the protein yield for each defatted flour. Crude protein content of LPI samples was approx. 90% (*m*/*m*) in the selected mung bean and cowpea varieties, while it was approx. 80% (*m*/*m*) in the black gram. Hence, the starting material, *i.e*. LPI samples used for the preparation of biopolymer particles had an acceptable level of purity (≥80%, *m*/*m*).

**Table 1:** Crude protein mass fraction of defatted flour (DF) and legume protein isolate (LPI) samples

Legume type	*w*(protein)_DF_/%	*w*(protein)_LPI_/%	*Y*(protein)/%
Mung bean MI6	26.5±0.7	87.3±0.3	52
Cowpea MI35	25.7±0.8	89.6±0.5	63
Black gram MI1	27.4±0.7	82.3±0.7	54

Protein mass fraction is shown as mean value±S.D. (*N*=3). See Eq. 1 for the calculation of *Y*

Protein yield (recovery) from the defatted flour samples was between 50 and 65% (*m*/*m*). This could be due to the poor solubility of legume proteins, which leads to an incomplete extraction. High molecular mass proteins present in legumes (*i.e*. legumins) are known to exhibit a lower solubility due to their presence in hexamer form at pH values below 10 and above 3 (*37*).

### Molecular mass profiles of LPI samples

**Fig S1:**
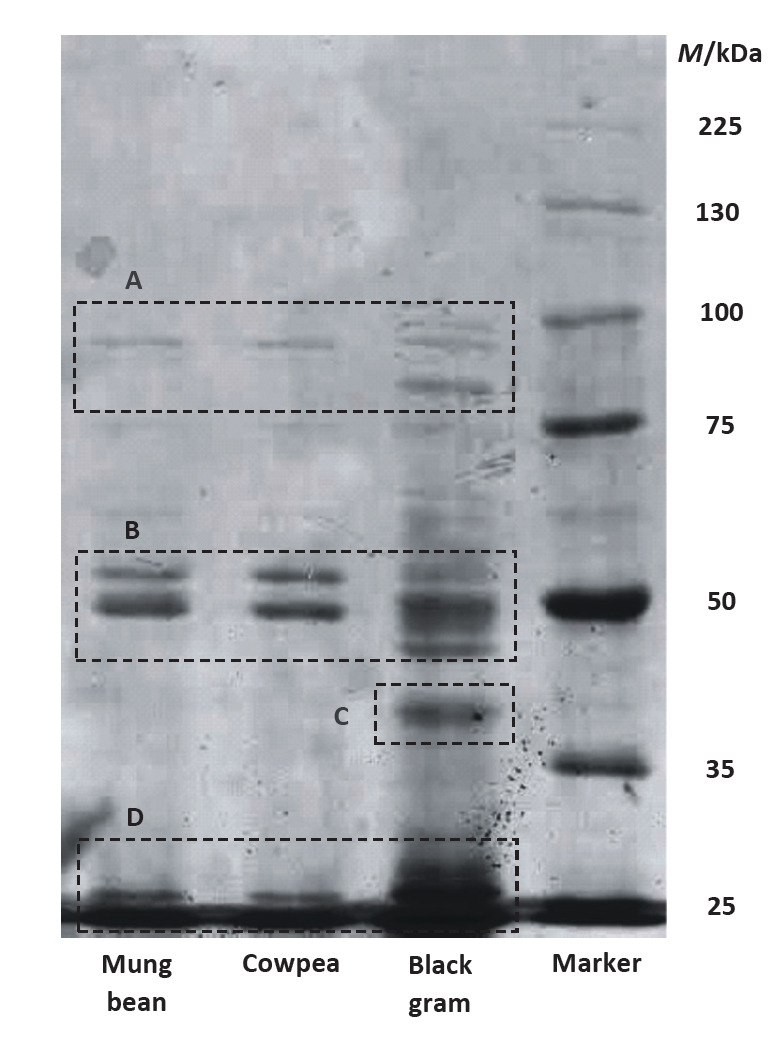
SDS-PAGE showing molecular mass profiles of legume protein isolate (LPI) samples. Electrophoresis was conducted at 150 V on a 12% resolving gel. LPI samples were reduced with β-mercaptoethanol prior to electrophoresis. The bands enclosed by the dashed rectangles are predicted to be resulting from dimeric forms (A), monomeric forms (B) and post-translationally modified forms (C and D) of vicilin subunits

An identical pattern of bands was observed in SDS-PAGE for the mung bean and cowpea LPI samples, with three intense bands near 55, 50 and 25 kDa, and a weaker band near 95 kDa suggesting that both had a similar protein/peptide composition (Fig. S1). The bands observed at ~55 and 50 were attributed to the presence of monomeric subunits of vicilins (*22*). The band observed at ~95 kDa was assumed to be resulting from dimeric subunits of vicilins. The absence of a band near 150 kDa for the trimeric vicilins and the low density observed of the band representing dimeric subunits suggested that trimeric vicilins present in mung bean and cowpea are prone to separate into their monomeric subunits, under the given extraction conditions. The ~25 kDa band might originate from post-translational proteolysis of monomeric subunits of vicilins (*38*).

Comparison of the band pattern of black gram LPI with mung bean and cowpea LPIs revealed that the molecular mass of the monomeric subunits of vicilins present in black gram LPI is distributed over a range of ~45-55 kDa, with prominent bands at ~45 and 50 kDa. The weaker bands observed at ~85-100 kDa were assumed to be resulting from dimeric subunits of vicilins. The bands observed at ~40 kDa and in the range of ~25-30 kDa were believed to result from post-translational proteolysis of monomeric subunits of black gram vicilins. The density of these bands suggested that proteolytic processing of black gram vicilins takes place at an extensive level, compared to mung bean and cowpea vicilins. Similarly, a higher susceptibility to post-translational proteolytic processing has been observed with monomeric subunits of vicilins present in pea (*Pisum sativum*) (*39*).

Subunits of black gram legumins may have also contributed to the ~40-kDa band (as the electrophoresis was conducted under reducing conditions), but given their lower solubility under the extracted conditions, their presence in the black gram LPI was thought to be considerably lower than in vicilins. Moreover, no ~40-kDa band was observed for the mung bean and cowpea LPI samples. Thus, all the prepared LPI samples were believed to mainly consist of vicilins. Lack of legumins explains the lower protein yield obtained from defatted flour samples (Table 1).

### ζ-Potential of individual biopolymer solutions

**Fig. 1:**
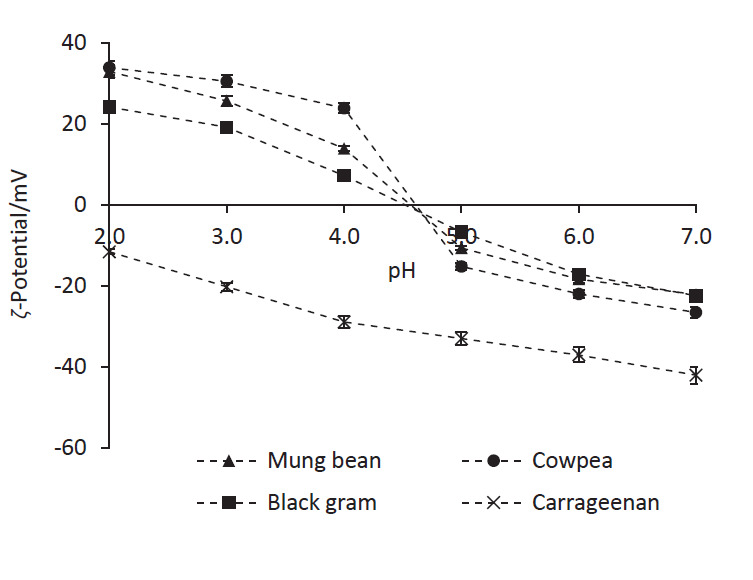
*ζ*-Potential of legume protein isolate samples and carrageenan solutions measured at different pH values. Biopolymer solutions of 0.1% (*m*/*V*) were used. Values are presented as mean±S.D. (*N*=3)

Fig. 1 shows how *ζ*-potential of individual biopolymer solutions deviates with pH. All the LPI samples showed a net positive charge at pH values below 4.5 and this was mainly attributed to the protonation of amine groups present in arginine and histidine amino acids, which have p*K*_a_ values of 12.5 and 6.0, respectively (*40*). When the pH was adjusted above 4.5, the LPI samples became negatively charged owing to the deprotonation of carboxyl groups present in aspartic and glutamic amino acids with p*K*_a_ values of 3.9 and 4.1, respectively (*40*). Isoelectric point (pI) of the LPI samples was determined by interpolation, and their pI values varied within a narrow pH range of 4.5 to 4.7 (Fig. 1). The pI values obtained for the LPI samples were found to be comparable with those of chickpea, lupine and lentil seed proteins (*41*).

It should be noted that as a given LPI contains more than one type of protein molecules (Fig. S1), the measured *ζ*-potentials indicate an average value derived from electrophoretic flow of all species preset in the LPI. The differences in the *ζ-*potential observed among the three LPI samples at a given pH can also be attributed to the differences in the types of protein present and/or their relative amounts. Carrageenan remained negatively charged within the experimented pH range due to the presence of sulfate groups attached to galactose and 3,6-anhydro-d-galactose monomer units. Magnitude of the negative charge of carrageenan increased with increasing pH, as the pH shift away from the p*K*_a_ of sulfate groups (*42*).

### Electrostatic interactions between LPI samples and carrageenan

**Fig. S2:**
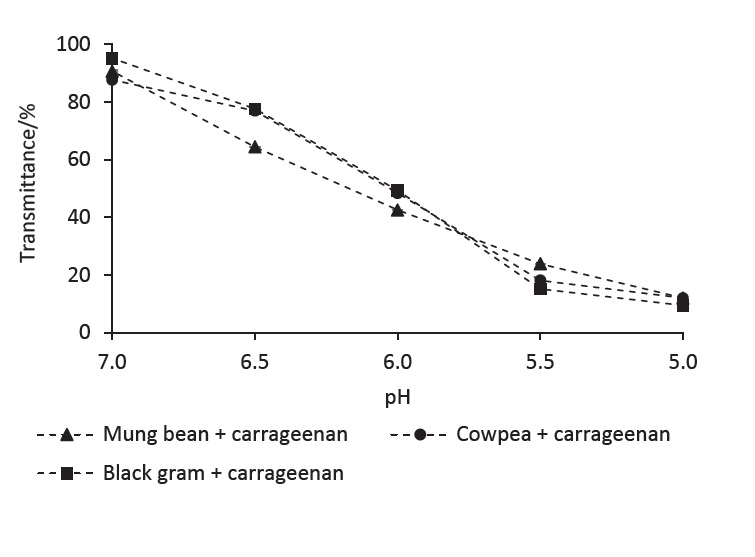
Transmittance of the mixtures at different pH values measured 1 h after preparation. Transmittance was measured at 600 nm. Mixtures contain legume protein isolate 0.1 and carrageenan 0.05% (*m*/*V*). Values are presented as mean±S.D. (*N*=3)

In order to facilitate electrostatic complex formation between LPI samples and carrageenan, individual solutions at pH=7 were mixed together, and then the pH of the mixtures was reduced step-wise up to pH=5. A decrease of transmittance (or an increase of turbidity) in the mixtures resembles the formation of biopolymer particles, and the transmittance in all three mixtures was progressively decreased with the reduction of pH (Fig. S2). Occurrence of electrostatic interactions between oppositely charged groups during biopolymer complex formation has been recently demonstrated by Rajabi *et al*. (*43*) and Gharanjig *et al*. (*20*) *via* Fourier transform infrared (FTIR) spectroscopy. IR absorption peaks that represent (*i*) stretching of charged groups (*e.g.* carboxylates, sulfates), (*ii*) stretching of C=O group in peptide bonds and (*iii*) stretching of C-N and bending of N-H bonds could shift or change their absorption intensity due to the formation of electrostatic complexes.

Reduction of pH results in an increased electrostatic potential difference between legume proteins and carrageenan, and this was evident by *ζ*-potential measurements (Fig. 1). Thus, the level of electrostatic interactions between legume proteins and carrageenan increases with the reduction of pH, resulting in more or larger biopolymer particles. The latter is more likely with the observed precipitation in the mixtures at lower pH values. Similar observations have been previously made by Hettiarachchi *et al*. (*44*). Transmittance of the individual solutions (*i.e*. three LPI samples and carrageenan) measured at the given pH values was always higher than those recorded for the mixtures (results not shown).

**Fig. 2:**
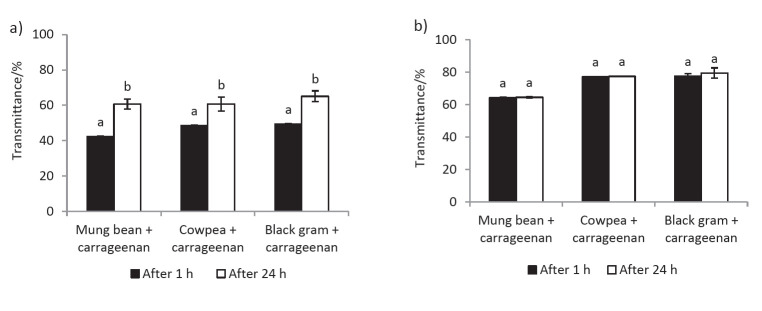
Transmittance of the mixtures measured 1 and 24 h after preparation: a) pH=6.0, and b) pH=6.5. Transmittance was measured at 600 nm. Mixtures contain legume protein isolate 0.1% and carrageenan 0.05% (*m*/*V*). Values are presented as mean±S.D. (*N*=3). For a given type of mixture, values assigned with different superscript letters are significantly different (p<0.05)

It should be noted that even though LPI samples showed a net negative *ζ*-potential within the experimented pH range (5.0-7.0), proteins present in these samples still carry some positively charged (or protonated) amine groups (to a lesser amount). The number of protonated groups present in legume proteins will increase with decreasing pH, facilitating more carrageenan molecules to electrostatically interact with them. Mixtures adjusted to pH=5 appeared turbid to the naked eye and after an hour they precipitated. Mixtures adjusted to pH=6.0 and 6.5 did not show any signs of precipitation within an hour. However, the mixtures at pH=6.0 showed a precipitate after 24 h, resulting in a significant increase (p<0.05) in transmittance (Fig. 2, a). In comparison, no precipitation took place in the mixtures at pH=6.5 after 24 h, and their transmittance values at 24 h were comparable (no significant difference) to those recorded 1 h after their preparation (Fig. 2b). Based on these results, we postulated that the biopolymer complexes formed in the mixtures at pH=6.5 are comparatively smaller in size and they carry adequate amount of charges on their surface (to repel each other) avoiding their aggregation and subsequent precipitation.

### Morphology of the biopolymer particles formed in selected mixtures

Aliquots obtained from the mixtures adjusted to pH=6.5 were subjected to SEM in order to observe the morphology of the biopolymer particles formed in those mixtures. SEM images were also obtained for the individual biopolymer solutions (three LPI samples and carrageenan) at the above pH value to serve as controls. No depositions were observed on the carbon tape for all the control samples (images not shown), confirming that the deposited particles observed for the mixtures resulted from the interactions between legume proteins and carrageenan, and they are not due to the self-aggregation of biopolymers.

**Fig. 3:**
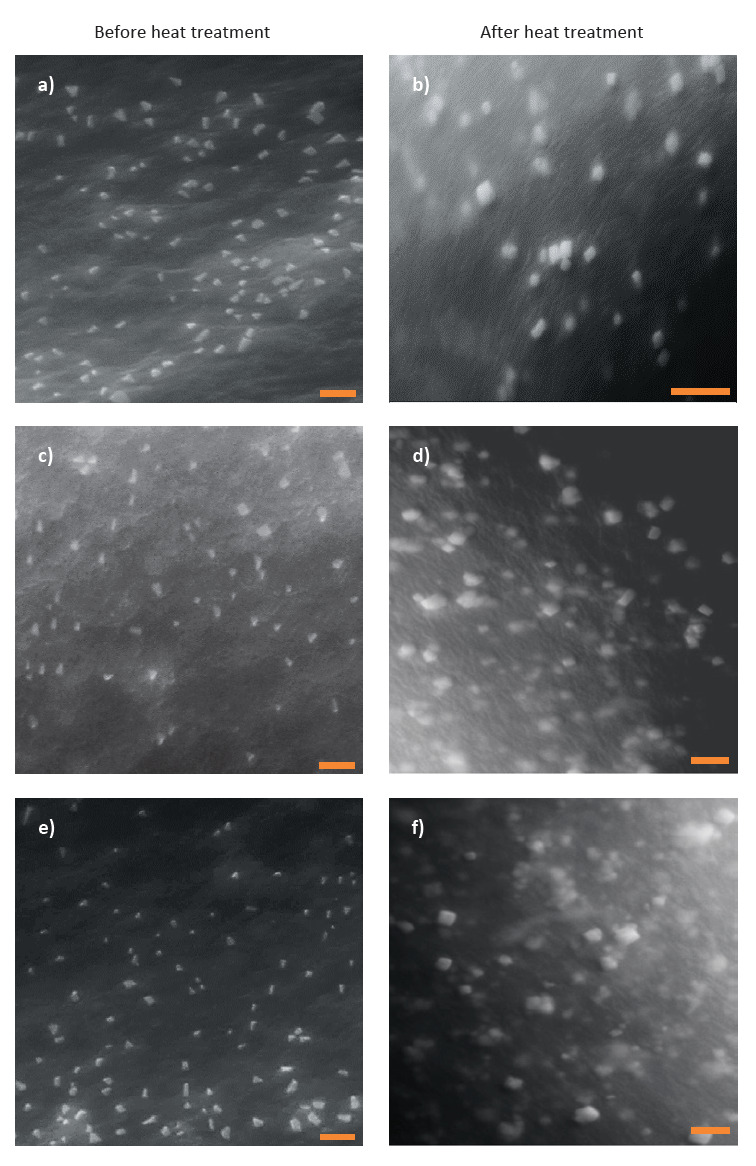
SEM images of biopolymer particles present in the mixtures at pH=6.5 before and after heat treatment (85 °C for 20 min): a) and b) mung bean+carrageenan, c) and d) cowpea+carrageenan, e) and f) black gram+carrageenan. Mixtures contain legume protein isolate 0.1% and carrageenan 0.05% (*m*/*V*). Scale bars represent 2.0 μm

The particles formed in all the selected mixtures appeared oblong or roughly spherical in shape (Fig. 3). When measuring the diameter of oblong biopolymer particles, the x-axis (*i.e*. short axis) was taken as the diameter. In each mixture, diameter of the biopolymer particles varied considerably over a broad range (Table 2), and this was attributed to the presence of different types of protein subunits in each LPI (Fig. S1). Availability of different types of protein subunits enables the formation of electrostatic complexes having different combinations of proteins with carrageenan, thus resulting in biopolymer particles of different sizes. Based on our observations, selection of an individual protein subunit or protein subunits within a narrow molecular mass range can be recommended to obtain biopolymer particles with a more uniform size.

**Table 2:** Average diameter of the biopolymer particles formed in legume protein isolate (LPI) and carrageenan mixtures at pH=6.5 before and after the heat treatment

LPI with carrageenan	*d*/nm	
Before heat treatment	After heat treatment
Mung bean	(117±32)^b^	(345±62)^a^
Cowpea	(138±40)^ab^	(343±91)^a^
Black gram	(154±59)^a^	(374±117)^a^

Diameter is given as mean value±S.D. (*N*=50). Within a column, diameters with different superscript letters are significantly different (p<0.05)

### Effect of heat treatment on the biopolymer particles

Heating the mixtures at pH=6.5 above the denaturation temperature of the legume proteins led to an increase in the diameter of the biopolymer particles. SEM images obtained for the heat-treated mixtures are given in Fig. 3. For all three mixtures, the average diameter of the biopolymer particles after the heat treatment was approximately two and half-fold greater than their average diameter before the heat treatment (Table 2). This was attributed to the unfolding of the proteins present in biopolymer particles. During the unfolding process, one possibility is that the carrageenan molecules linked to the proteins may become detached, and the unfolded protein molecules will interact with each other resulting in larger biopolymer particles that are mainly composed of proteins (*12*, *13*). Aggregation of unfolded protein molecules can take place *via* the formation of intermolecular disulfide bonds and increased hydrophobic interactions (*45*).

**Fig. 4:**
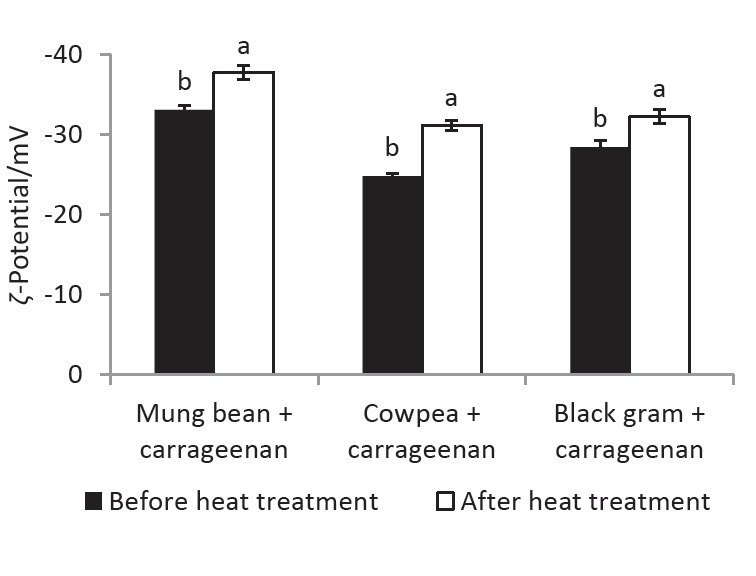
*ζ*-Potential of the biopolymer particles present in the mixtures at pH=6.5 before and after heat treatment (85 °C for 20 min). Mixtures contain legume protein isolate 0.1% and carrageenan 0.05% (*m*/*V*). Values are presented as mean±S.D. (*N*=3). For a given type of mixture, values with different superscript letters are significantly different (p<0.05)

Alternatively, the unfolded protein subunits still partially attached to carrageenan molecules can aggregate (*9*), thus facilitating electrostatic attachment of more carrageenan molecules due to the exposure of new interaction sites, resulting in the formation of larger biopolymer particles with a higher number of both protein and carrageenan molecules. The significant increase (p<0.05) in the net negative surface charge observed for the larger biopolymer particles after the heat treatment (Fig. 4) suggests that the latter scenario is more plausible for the biopolymer particles formed during this study. If the biopolymer particles formed after the heat treatment are mainly composed of protein molecules, it is unlikely to observe an increase in their net negative surface charge.

**Fig. S3:**
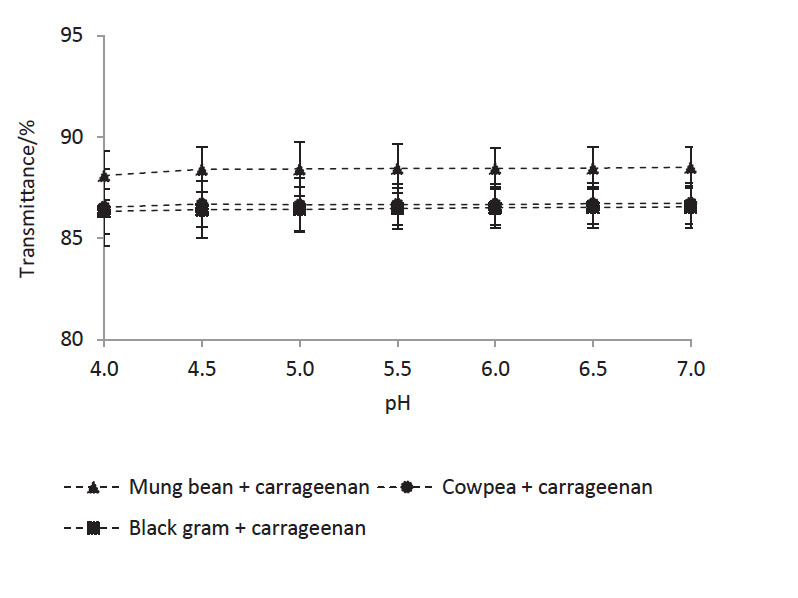
Transmittance of the heat-treated mixtures at different pH values measured 1 h after preparation. Transmittance was measured at 600 nm. Mixtures contain legume protein isolate 0.02 and carrageenan 0.01% (*m*/*V*). Values are presented as mean±SD (*N*=3)

After the heat treatment, the transmittance of a given mixture remained stable with the change of pH, suggesting that biopolymer particles in the mixtures were less prone to aggregation even at pH values below the pI of the LPI (Fig. S3), enabling them to be used as stable colloidal systems in food applications. This was attributed to the increased negative surface charge of the biopolymer particles (Fig. 4), and it is possible that carrageenan molecules occupy a higher proportion of their surface, keeping them repelled from each other even at lower pH values. In addition, heat-induced Maillard reaction between free amino groups of LPIs and terminal reducing sugar moieties of carrageenan could lead to form covalently bound conjugates (*46*), increasing the structural stability of the biopolymer particles against pH-induced dissociation and re-association. Formation of such covalent bonds in soy protein isolate-carrageenan biopolymer particles has been previously reported by Mao *et al*. (*47*) based on the modifications observed in C=O and C-N stretching absorption peaks of the IR spectrum. Further studies will be conducted on these LPI-carrageenan biopolymer particles in the future to elucidate their composition, structure and underlying interactions.

## CONCLUSIONS

Mung bean, cowpea and black gram legume protein isolate (LPI) samples mainly consisted of monomeric (~50 kDa) and dimeric (~100 kDa) subunits, and post-translationally modified forms (<50 kDa) of 7S vilcilins. These LPI samples were capable of forming stable biopolymer particles with carrageenan at pH=6.5 by limited electrostatic interactions. The average diameter of these biopolymer particles was in the range of ~115-155 nm, and they had a negative surface charge of ~28-33 mV, which avoided their aggregation. Application of heat (85 °C for 20 min) increased the average diameter of the biopolymer particles (~345-375 nm), and this was attributed to the unfolding and aggregation of legume proteins. The heat-treated biopolymer particles had a higher negative surface charge at pH=6.5 and they were resistant to aggregation over a broader pH range (4.0-7.0). It could be also suggested that formation of Maillard conjugates during the heat treatment may have led to increase the stability of the biopolymer particles.
